# Prognostic analysis of elderly patients with pathogenic microorganisms positive for sepsis-associated encephalopathy

**DOI:** 10.3389/fmicb.2024.1509726

**Published:** 2024-12-16

**Authors:** Xiaopeng Shi, Lijun Xu, Lijuan Jing, Zehua Wang, Lina Zhao, Xiangmei Zhao

**Affiliations:** ^1^Department of Emergency, Henan Provincial People's Hospital, Zhengzhou University People's Hospital, Zhengzhou, Henan, China; ^2^Department of Critical Care Medicine, Tianjin Medical University General Hospital, Tianjin, China

**Keywords:** sepsis, sepsis-associated encephalopathy, prognosis, SAPS III, pathogenic microorganisms

## Abstract

**Objectives:**

Sepsis-associated encephalopathy (SAE) has a high incidence and mortality, especially for elderly patients and patients who are positive for pathogenic microbial infection, this study explored the prognostic factors influencing the prognosis of elderly patients with pathogenic microorganisms positive of sepsis-associated encephalopathy.

**Methods:**

Patients with SAE and pathogenic microbiology positive were included in this study from Medical Information Mart for Intensive Care IV (MIMIC IV) database. The main results of this study was analyzed the 28-day mortality rate of patients with pathogenic microorganism positive and SAE by Wilcoxon, Kaplan–Meier curve and other methods.

**Results:**

This study found that older patients with SAE had higher mortality at 28 and 90 days compared with non-older patients with SAE. *Klebsiella pneumoniae* and *Pseudomonas aeruginosa* infection, the level of APTT and lactate and SAPS III score were independent risk factors for 28-day mortality in elderly patients with SAE, among them, *Klebsiella pneumoniae* and *Pseudomonas aeruginosa* infection had the best sensitivity (0.893; 0.931) in assessing elderly patients with pathogenic microorganisms positive and SAE; the SAPS III score had the highest AUC (0.681) value and specificity (0.761) in assessing elderly patients with pathogenic microorganisms positive and SAE.

**Conclusion:**

The older patients with SAE had a poor prognosis, the elder patients with pathogenic microorganisms positive and SAE with high levels of APTT and lactate and SAPS III score and *Klebsiella pneumoniae* and *Pseudomonas aeruginosa* infection should be closely monitored and treated aggressively.

## Introduction

Sepsis-associated encephalopathy (SAE) is a serious complication in the intensive care unit (ICU) in sepsis, and its has high morbidity and mortality rate, and long-term cognitive and functional impairment bring a heavy burden to the patient’s family and society (Bircak-Kuchtova et al., 2023; Ehler et al., 2017; Zhao et al., 2021; Ura et al., 2022; Yu et al., 2024). As the population ages, the disease has become an important issue in the field of public health.

In recent years, the research on SAE in elderly sepsis patients have been increasingly deepened at home and abroad (Manabe and Heneka, 2021; Aronsson Dannewitz et al., 2024; Barrett et al., 2024). Especially, many previous studies had found that patients with sepsis infected with pathogenic microorganisms such as *Klebsiella pneumoniae* and *Acinetobacter baumannii* increased multi-organ impairment and mortality in sepsis (Hosoda et al., 2021; Zilberberg et al., 2016; Todi et al., 2024; Arbous et al., 2024). The studies have shown that SAE has a high incidence and poor prognosis in elderly patients with sepsis, which seriously affects the quality of life and survival of patients, the mortality rate in patients with sepsis encephalopathy is about 50–70%. Long-term follow-up showed that about 45% of patients with sepsis had cognitive dysfunction such as inattention and memory loss 1 year after discharge, which seriously affected the quality of life of patients (Feng et al., 2017; Chen et al., 2020; Pakvasa et al., 2017; Gofton and Young, 2012). The current research focuses on mechanisms such as inflammatory response, neurotransmitter dysregulation, and blood–brain barrier damage, as well as exploring novel biomarkers and therapeutics to improve the prognosis of patients with SAE (Zhao et al., 2024; Zhao et al., 2022). The relationship between the type of pathogenic microorganisms infection, in particular, the common of *Klebsiella pneumoniae*, *Acinetobacter baumannii*, *Pseudomonas aeruginosa*, etc. and the prognosis of patients with SAE remain unclear.

Therefore, the purpose of this study was to analyze the prognostic factors of elderly patients with SAE and to identify the key indicators affecting the prognosis of SAE patients through retrospective analysis of data, such as pathogenic species, source of infection, and underlying disease status. The hypothesis is that the prognosis of elderly patients with SAE is closely related to the type of pathogenic microorganisms, the site of infection by analysis of SAE patients who are positive for pathogenic microorganisms, and it is expected to improve the prognosis of such patients by optimizing the treatment regimen and strengthening the management of the underlying diseases.

## Materials and methods

### Patient

The patients in this study were from elderly patients with pathogenic microbial-positive SAE from 2008 to 2019 in the Medical Information Mart for Intensive Care IV (MIMIC IV) database hospital (Johnson et al., 2023). Screening criteria include: (1) Age ≥ 18 years; (2) Diagnosed with sepsis 3.0 and positive for pathogenic microorganisms (Singer et al., 2016); (3) SAE as defined and with reference to previous studies, in this study, SAE was defined as: sepsis with a Glasgow Coma Scale (GCS) < 15 during ICU hospitalization, or they were diagnosed as: delirium, cognitive impairment, altered mental status according to the ICD-9 code, or medicating with haloperidol (Zhao et al., 2021; Sonneville et al., 2023; Eidelman et al., 1996); and (4) Exclude encephalopathy caused by other central nervous system diseases (traumatic brain injury, intracerebral hemorrhage, cerebral embolism, ischemic stroke, epilepsy, or intracranial infection and another cerebrovascular disease, mental disorders, and neurological disease, chronic alcohol or drug abuse, metabolic encephalopathy, hepatic encephalopathy, hypertensive encephalopathy, hypoglycemic coma, and other liver disease or kidney disease affecting consciousness) (Peng et al., 2022).

### Data collection

In this study, the clinical data of sepsis patients with pathogenic microorganisms positive were collected from the MIMIC IV database, including the basic information of the patients (age, male), the detection results of pathogenic microorganisms (*Acinetobacter baumannii, Klebsiella pneumoniae, Pseudomonas aeruginosa, Pseudomonas aeruginosa, Staphylococcus aureus, Escherichia coli*), the site of infection, comorbidities (hypertension, diabetes, chronic obstructive pulmonary disease, Chronic kidney disease), the worst value of vital signs and laboratory tests within 24 h of admission, the worst disease severity score of Sequential Organ Failure Assessment score (SOFA), Simplified Acute Physiology Score II (SAPS II), SAPS III, model for end-stage liver disease (MELD), Logistic Organ Dysfunction System (LODS), Oxford acute severity of illness score (OASIS) were recorded during ICU hospitalization. Besides, we searched for vasoactive drugs and renal replacement therapy during hospital stays, the prognostic indicators of ICU admission, length of hospitalization, 28-day mortality rate and 90-day mortality rate were recorded in this study. In this study, the entire data retrieval and integration were carried out using SQL language and R language.

### Statistics

The continuous variables in this study were all skewed, which were presented as the interquartile range (IQR). Since the study does not satisfy the normal distribution, the Wilcoxon rank-sum and Fisher’s exact tests were used for the comparison of elderly patients with SAE versus non-elderly patients with SAE, and survival group patients versus non-survival group patients. To analyze the relationship between covariates and 28-day mortality in elderly patients with SAE, univariate and multivariate COX regression analyses were selected. The covariates explored were used to assess the prognostic performance of older patients with SAE, using ROC curves. The section assesses the discrimination of indicators by plotting the receiver operating characteristic (ROC) curve and calculating the area under the curve (AUC) to determine the predictive accuracy of biomarkers for the prognosis of elderly patients with pathogenic microorganism-positive and SAE. In order to avoid bias due to multiplicity, we applied Bonferroni for correction, and the *p*-value given in this study is the corrected *p*-value. The Kaplan–Meier (KM) curves were used to analyze the prognosis of mortality at 28 days and 90 days in elderly and non-elderly patients.

### Outcome

#### The baseline data of patients with pathogenic microorganism-positive and sepsis-associated encephalopathy

A total of 5,694 sepsis patients with pathogenic microorganism-positive were included in this study, among them, 2,896 sepsis patients were diagnosed with SAE according to the diagnostic criteria for SAE, a total of 1,296 patients were excluded according to the exclusion criteria excluded patients include: intracerebral hemorrhage, cerebral embolism, and ischemic stroke (*n* = 654), traumatic brain injury (*n* = 65), meningitis and encephalitis (*n* = 97), other cerebrovascular diseases (*n* = 134), mental disorders and neurological disease (*n* = 103), chronic alcohol or drug abuse (*n* = 189), metabolic encephalopathy (*n* = 31), hepatic encephalopathy (*n* = 23). and 1,600 patients were finally included in the analysis, there were 1,008 patients in the elderly SAE group and 592 patients in the non-elderly SAE group.

The results of the study in Table 1 showed that compared with non-older patients with SAE, the Charlson score of SAE was higher in pathogenic microorganism positive, and more patients were diagnosed with hypertension, diabetes, chronic obstructive pulmonary disease, chronic kidney disease, urinary tract infection, *Staphylococcus aureus*, and *E. coli* infections. Compared with non-older patients with SAE, the older patients with SAE had higher levels of temperature, creatinine and bun, and a faster of respiratory rate, a lower of systolic and diastolic blood pressure, hemoglobin, and oxygen saturation levels. The results of the prognostic study showed that older patients with SAE were more severely ill and had higher LODS, and OASIS scores than non- older patients with SAE.

**Table 1:** Baseline and outcome of sepsis-associated encephalopathy.

Characteristic	Non-elderly patients group (*n* = 592)	Elderly patients group (*n* = 1,008)	*p*
Age, years	55.00 [47.00, 61.00]	77.00 [71.00, 84.00]	<0.001
Male sex, *n* (%)	346 (58.4)	530 (52.6)	0.026
Co-morbid conditions, *n* (%)			
Charlson	3.00 [2.00, 5.00]	6.00 [4.75, 8.00]	<0.001
Hypertension	251 (42.4)	508 (50.4)	0.002
Diabetes	158 (26.7)	367 (36.4)	<0.001
Chronic obstructive pulmonary disease	109 (18.4)	289 (28.7)	<0.001
Chronic kidney disease	100 (16.9)	316 (31.3)	<0.001
Site of infection, *n* (%)			
Pulmonary infection	52 (8.8)	93 (9.2)	0.836
Abdominal infection	31 (5.2)	49 (4.9)	0.831
Urinary infection	37 (6.2)	112 (11.1)	0.002
Skin soft tissue infection	30 (5.1)	67 (6.6)	0.242
Catheter infection	25 (4.2)	34 (3.4)	0.463
Pathogenic microorganisms, *n* (%)			
*Acinetobacter baumannii*	13 (2.2)	14 (1.4)	0.313
*Klebsiella pneumoniae*	83 (14.0)	150 (14.9)	0.691
*Pseudomonas aeruginosa*	57 (9.6)	122 (12.1)	0.152
*Staphylococcus aureus*	42 (7.1)	110 (10.9)	0.015
*E. coli*	105 (17.7)	239 (23.7)	0.006
Physiology			
Temperature, °C	37.39 [37.00, 37.83]	37.17 [36.89, 37.56]	<0.001
Heart rate, beats per minute	93.00 [80.75, 107.00]	90.00 [77.75, 106.00]	0.043
Systolic blood pressure, mmHg	107.00 [94.00, 126.00]	105.00 [90.00, 123.00]	0.02
Diastolic blood pressure, mmHg	59.00 [50.00, 70.00]	52.00 [43.00, 61.00]	<0.001
Respiratory rate, beats per minute	22.00 [17.00, 27.00]	22.75 [18.00, 27.00]	0.012
Laboratory tests			
Blood system			
White blood cell×10^9^/L	13.60 [9.40, 18.48]	13.40 [9.40, 17.60]	0.607
Hemoglobin (g/dL)	9.60 [8.10, 11.40]	9.30 [7.90, 10.90]	0.005
Platelet (×10ˆ9/L)	166.50 [107.00, 241.75]	177.00 [118.00, 236.00]	0.184
PT (sec)	14.90 [12.88, 18.80]	15.40 [13.10, 18.80]	0.231
APTT (sec)	35.00 [29.08, 45.62]	34.30 [29.30, 44.78]	0.938
INR	1.40 [1.20, 1.72]	1.40 [1.20, 1.72]	0.231
Other organ functions			
Glasgow Coma Scale	15.00 [14.00, 15.00]	15.00 [14.00, 15.00]	0.075
Creatinine (mg/dL)	1.00 [0.70, 1.80]	1.20 [0.90, 1.90]	<0.001
Bun (mg/dL)	19.00 [13.00, 32.00]	26.00 [18.00, 42.00]	<0.001
Glucose (mg/dL)	160.00 [126.00, 202.00]	163.00 [130.00, 207.00]	0.589
Lactate (mmol/L)	2.10 [1.30, 2.50]	2.10 [1.30, 2.50]	0.697
PaCO_2_, mmHg	40.00 [35.00, 45.00]	41.00 [35.00, 45.00]	0.352
SpO_2_, %	93.00 [91.00, 95.00]	92.50 [90.00, 95.00]	0.002
Treatment strategies			
Use of vasoactive drugs, *n* (%)	224 (37.8)	414 (41.1)	0.222
Renal replacement therapy, *n* (%)	44 (7.4)	52 (5.2)	0.082
Outcome			
SOFA	3.00 [2.00, 5.00]	3.00 [2.00, 5.00]	0.067
SAPS II			
SAPS III	46.00 [34.00, 62.00]	48.00 [38.00, 61.00]	0.045
MELD	11.00 [8.00, 23.00]	13.89 [9.00, 20.39]	0.204
LODS	5.00 [3.00, 7.00]	5.00 [3.00, 7.00]	0.001
OASIS	31.50 [26.00, 37.00]	34.00 [29.00, 39.00]	<0.001
Los_icu	4.24 [2.08, 10.07]	3.59 [1.93, 7.16]	0.001
Los_hospital	14.84 [7.45, 26.88]	11.87 [6.98, 20.74]	<0.001

APPT, activated partial thrombin time; BUN, blood urea nitrogen; INR, international normalized ratio; PT, prothrombin time; SAPS, simplified acute physiology score; SOFA, sequential organ failure assessment; LODS, logical evaluation system for organ dysfunction; OASIS, Oxford acute severity of illness score; *p* < 0.05, statistically significant.

#### The prognosis of patients with pathogenic microorganism-positive and sepsis-associated encephalopathy

**Figure 1:**
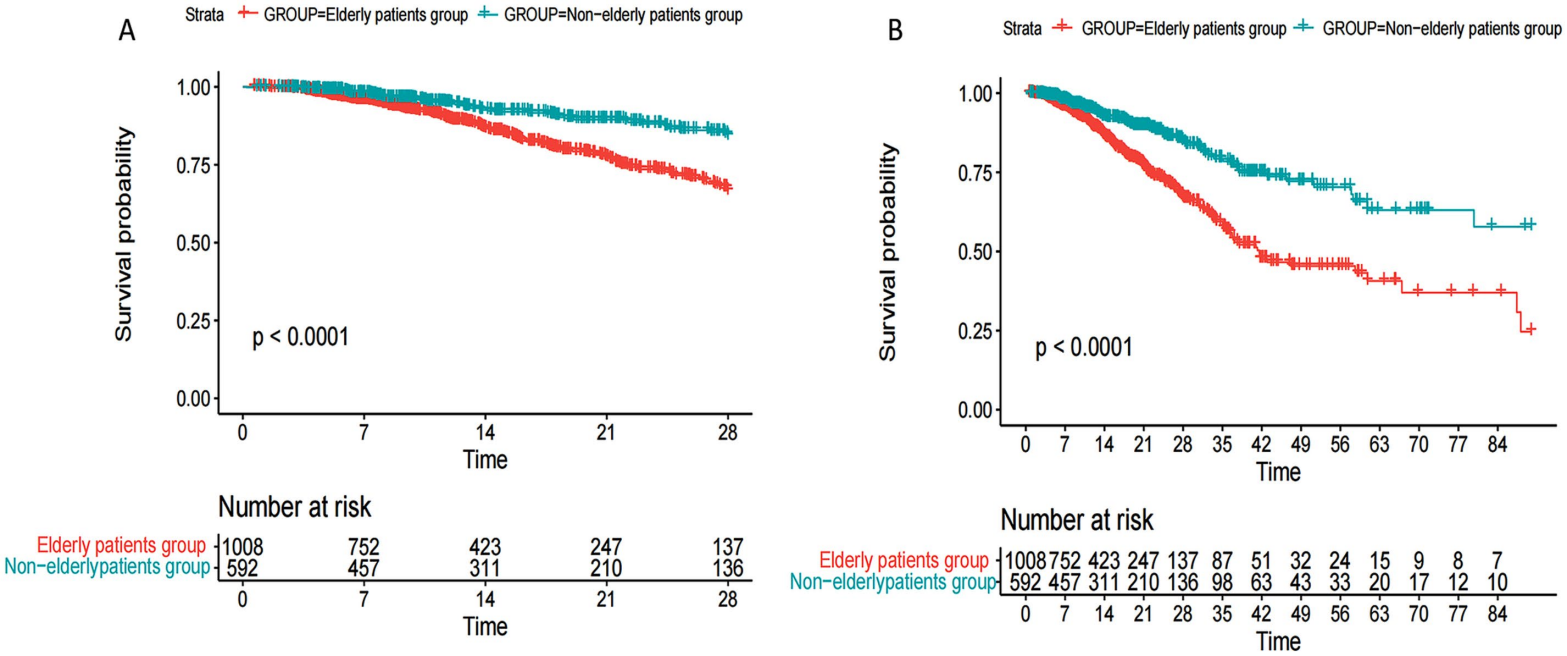
Kaplan–Meier curves of 28-day and 90-day mortality in elderly and non-elderly sepsis-associated encephalopathy. **(A)** Comparison of 28-day mortality in elderly versus non-elderly patients with SAE; **(B)** Comparison of 90-day mortality in elderly versus non-elderly patients with SAE.

Figure 1 compares the prognosis of older patients with SAE and non-older with SAE, the results showed that the 28-day and 90-day mortality rates of elderly patients with SAE were significantly higher than those of non-elderly patients with SAE through the KM curve.

#### Univariate and multivariate COX regression analysis in elderly patients with pathogenic microorganism positive and sepsis-associated encephalopathy

According to the results of Table 1 and Figure 1, univariate and multivariate COX regression analyses were performed in Table 2 for elderly patients with pathogenic microorganisms positive and SAE. Table 2 showed that *Klebsiella pneumoniae* and *Pseudomonas aeruginosa* infection, the level of activated partial thrombin time (APTT) and lactate and SAPS III were independent risk factors for 28-day mortality in elderly patients with SAE. Besides, the study of supplementary material 1 found that the common types of infection with pathogenic microorganisms in the ICU were not associated with the prognosis of patients with non- elderly sepsis-associated encephalopathy.

**Table 2:** Univariate and multivariate COX regression analysis for sepsis-associated encephalopathy.

	Univariate				Multivariate			
Characteristic	HR	95% CI		*p*	HR	95% CI		*p*
		Lower	Upper			Lower	Upper	
Age, years	1.020	1.000	1.040	0.05				
Male sex	0.921	0.675	1.257	0.60				
Charlson	1.068	1.003	1.138	0.04	1.061	0.993	1.133	0.08
Hypertension	0.843	0.615	1.156	0.29				
Diabetes	0.766	0.548	1.070	0.12				
Chronic obstructive pulmonary disease	1.102	0.778	1.561	0.58				
Chronic kidney disease	1.090	0.785	1.513	0.61				
Pulmonary infections	1.012	0.612	1.674	0.96				
Abdominal infections	0.483	0.179	1.303	0.15				
Urinary infections	0.868	0.501	1.503	0.61				
Skin soft tissue infections	1.448	0.863	2.427	0.16				
Catheter infections	0.801	0.354	1.812	0.59				
*Acinetobacter baumannii*	0.801	0.198	3.233	0.76				
*Klebsiella pneumoniae*	1.779	1.075	2.944	0.03	2.225	1.318	3.757	<0.001
*Pseudomonas aeruginosa*	2.114	1.145	3.902	0.02	2.178	1.162	4.083	0.020
*Staphylococcus aureus*	0.815	0.471	1.410	0.46				
*E. coli*	0.813	0.552	1.198	0.30				
Temperature	1.005	0.806	1.253	0.97				
Heart rate	1.007	1.000	1.014	0.06				
Systolic blood pressure	0.994	0.988	1.001	0.09				
Diastolic blood pressure	0.988	0.977	1.000	0.05				
Respiratory rate	1.023	1.000	1.045	0.05				
White blood cell	0.999	0.985	1.012	0.83				
Hemoglobin	1.054	0.983	1.131	0.14				
Platelet	1.000	0.998	1.001	0.61				
PT	1.009	1.000	1.018	0.06				
APTT	1.006	1.003	1.010	<0.001	1.005	1.000	1.009	0.030
INR	1.079	0.991	1.175	0.08				
Glasgow Coma Scale	0.932	0.869	0.999	0.05				
Creatinine	1.079	0.979	1.188	0.12				
Bun	1.005	0.999	1.010	0.08				
Glucose	1.000	0.998	1.002	0.88				
Lactate	1.125	1.065	1.189	<0.001	1.083	1.016	1.154	0.01
PaCO_2_	0.997	0.982	1.012	0.71				
SpO_2_	0.985	0.969	1.002	0.08				
Use of vasoactive drugs	1.662	1.209	2.283	<0.001	1.354	0.965	1.899	0.08
CRRT	1.144	0.648	2.022	0.64				
SOFA	1.033	0.964	1.106	0.36				
SAPS II	1.024	1.013	1.036	<0.001	1.008	0.994	1.023	0.25
SAPS III	1.019	1.013	1.026	<0.001	1.014	1.006	1.023	<0.001
LODS	1.122	1.067	1.180	<0.001	1.009	0.944	1.077	0.80
OASIS	1.026	1.008	1.045	<0.001	1.000	0.979	1.022	0.98

APPT, activated partial thrombin time; BUN, blood urea nitrogen; INR, international normalized ratio; PT, prothrombin time; SOFA, sequential organ failure assessment score; CRRT, continuous renal replacement therapy; SAPS II, simplified acute physiology score; LODS, logistic organ dysfunction system score; OASIS, Oxford acute severity of illness score. *p* < 0.05, statistically significant.

#### The ROC curves, specificity and sensitivity analysis of independent risk factor indicators of 28-day mortality in elderly patients with sepsis-associated encephalopathy

**Figure 2:**
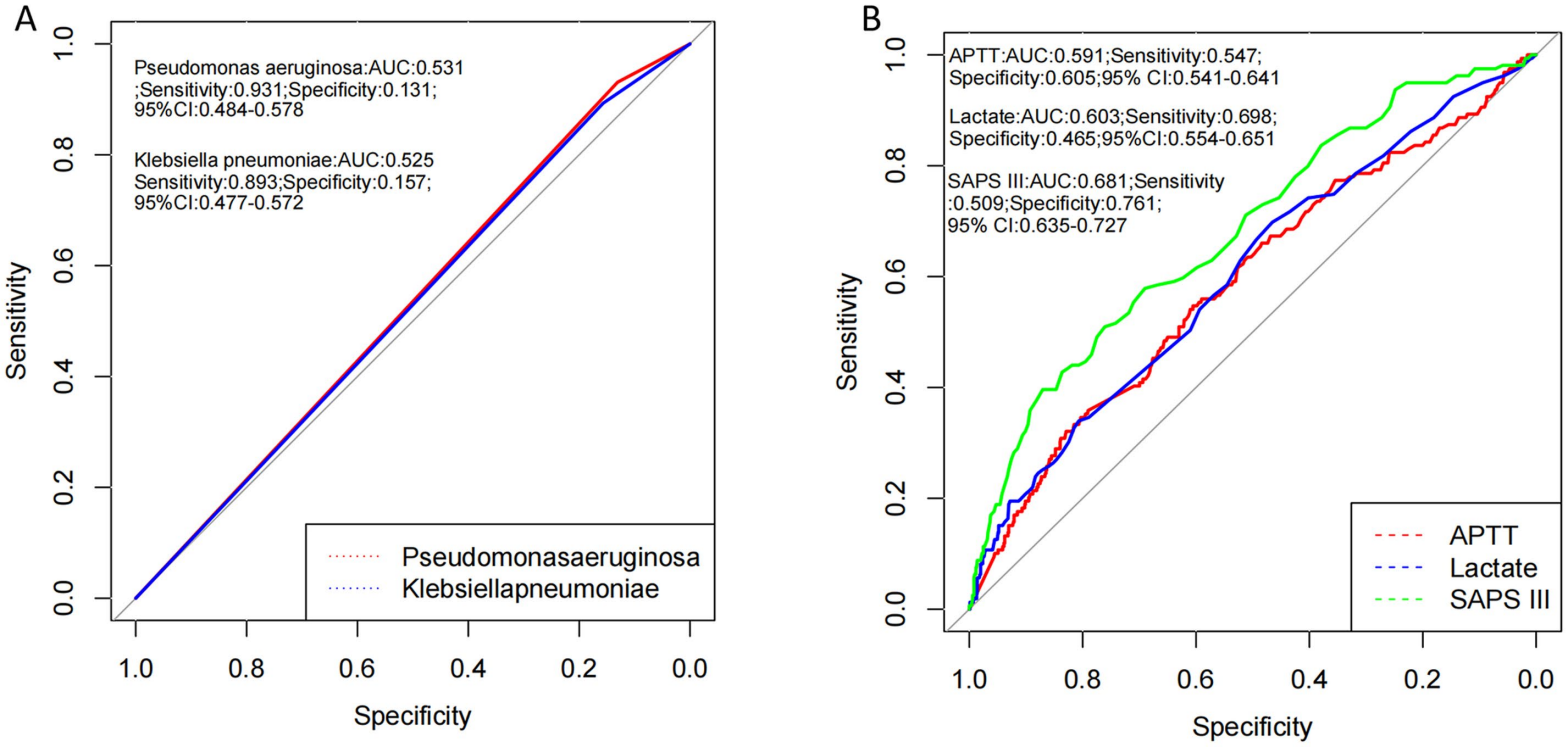
Analysis of the AUC values, specificity and sensitivity of *Pseudomonas aeruginosa*, *Klebsiella pneumoniae*, the levels of lactate, APTT, SAPS III scores in the area under the ROC curve of 28-day mortality in elderly sepsis patients with pathogenic microorganisms positive and SAE. This figure shows that the AUC and specificity of SAPS III score was the better than other indicators in the prognosis of SAE, the sensitive *of Pseudomonas aeruginosa* and *Klebsiella pneumoniae* infection were the better than other indicators in the prognosis of SAE. SAPS III, simplified acute physiology score; APPT, activated partial thrombin time.

Figure 2 shows the ROC curve, specificity, and sensitivity of *Klebsiella pneumoniae* and *Pseudomonas aeruginosa* infection, APTT and lactate, and SAPS III score for 28-day mortality in elderly SAE. The results of the study of Figure 2 shows that the AUC of *Klebsiella pneumoniae*, *Pseudomonas aeruginosa*, APTT and lactate, and SAPS III score, respectively, were 0.525, 0.531, 0.591, 0.603, 0.681; the sensitivity of *Klebsiella pneumoniae*, *Pseudomonas aeruginosa*, APTT and lactate, and SAPS III score, respectively, were 0.893, 0.931, 0.547, 0.698, 0.509; the specificity of *Klebsiella pneumoniae*, *Pseudomonas aeruginosa*, APTT and lactate, and SAPS III score, respectively, were 0.157, 0.131, 0.465, 0.605, 0.761; the AUC and specificity of SAPS III score was the better than other indicators in the prognosis of elderly patients with pathogenic microorganisms positive and SAE, while the sensitive *of Pseudomonas aeruginosa* and *Klebsiella pneumoniae* infection were the better than other indicators.

#### The levels of APTT, lactate, and SAPS III score were compared in the 28-day and 90-day mortality of elderly patients with pathogenic microorganism-positive and sepsis-associated encephalopathy

**Figure 3:**
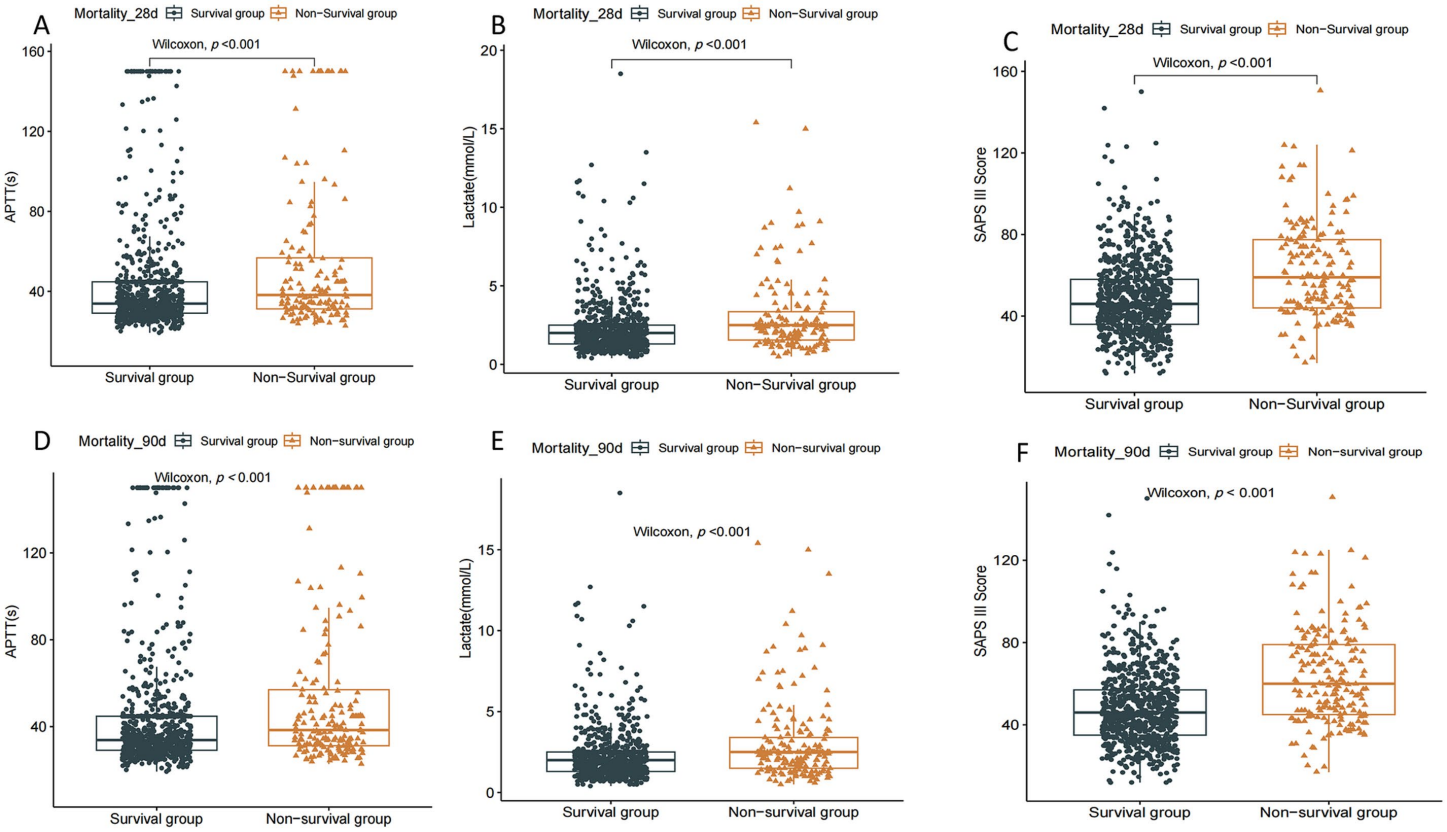
The levels of lactate, APTT, and SAPS III scores were compared in 28-day versus 90-day mortality in elderly sepsis patients with pathogenic microorganisms positive and sepsis-associated encephalopathy. SAPS III, simplified acute physiology score; APPT, activated partial thrombin time.

The results of Figure 3 showed that the levels of APTT, lactate, and SAPS III scores were significantly higher than those in the 90-day and 28-day of elderly sepsis patients with positive for pathogenic microorganisms and SAE (*p* < 0.001).

## Discussion

This study conducted an in-depth analysis of the prognosis of elderly sepsis patients with pathogenic microorganism-positive and SAE. The results showed that the prognosis of elderly sepsis patients with SAE was generally poor, and their high mortality rate was closely related to the severity of SAE. Further analysis found that *Klebsiella pneumoniae* and *Pseudomonas aeruginosa* infection, the high level of APTT and lactate and SAPS III score were the main factors contributing to poor prognosis. The results of this study suggest that early identification and intervention of SAE and optimization of ICU management strategies were great significance for improving the prognosis of sepsis elderly patients.

Previous studies had found that sepsis-associated encephalopathy is as high as approximately 50–70%, the mortality rate of patients with sepsis who progress to SAE is about 10–50% (Sonneville et al., 2023; Huang et al., 2021; Zhang et al., 2023). The results of this study suggest that among the patients with sepsis-associated encephalopathy, 63% of the elderly patients with sepsis-associated encephalopathy, the mortality rate of elderly patients with SAE was significantly higher than that of non-elderly patients, this study found that the mortality rate of patients with SAE was about 20%, which is consistent with previous studies. Although specific morbidity and mortality vary depending on diagnostic criteria, underlying patient status, and treatment, the general consensus is that SAE significantly increases the risk of mortality and long-term cognitive impairment in older patients with sepsis (Gu et al., 2023; Tsuruta and Oda, 2016). This study further emphasizes the importance of early screening, aggressive control of primary infection, and optimal management of sepsis in order to improve prognosis in sepsis older patients.

The prognosis of elderly patients with pathogenic microorganism-positive and SAE is multifactorial. Major independent risk factors include *Klebsiella pneumoniae* and *Pseudomonas aeruginosa* infection, the high level of APTT and lactate and SAPS III score, *Klebsiella pneumoniae* and *Pseudomonas aeruginosa* infection, the high level of APTT and lactate, and SAPS III score significantly increased the risk of mortality. Identification of these risk factors is helpful for early clinical intervention and optimization of treatment strategies, thereby improving the prognosis of elderly patients with SAE.

As a tool to predict the mortality rate of ICU patients, the SAPS III score also has important application value in patients with sepsis (Hou et al., 2020; Zhu et al., 2022). The scoring system can accurately predict the mortality risk of patients with sepsis by comprehensively evaluating the physiological indicators, age and underlying diseases of patients, and provide a basis for clinical decision-making and medical resource allocation. Its simplicity and speed make the SAPS III score widely used in the initial evaluation and monitoring of patients with sepsis. In this section, a retrospective study found a significant correlation between SAPS III score and mortality in older patients with SAE. The SAPS III score can effectively predict the prognosis of elderly patients with pathogenic microorganisms positive and SAE, and the higher the score, the 28 days mortality rate with sepsis patients is significantly increased. These results suggest that the SAPS III score can be used as an important tool to clinically assess the severity and predict mortality of sepsis elderly patients with pathogenic microbial-positive and SAE.

Common pathogens that cause sepsis in intensive care medicine include: *E. coli*, *Klebsiella pneumoniae* and *Pseudomonas aeruginosa* in gram-negative bacteria; Gram-positive bacteria include *Staphylococcus aureus*, the release of cell wall components and exotoxins from these bacteria can cause a systemic inflammatory response syndrome, leading to organ dysfunction, and the mortality rate of sepsis patients increases significantly as the number of organs affected. In this study, it was found that *Klebsiella pneumoniae* and *Pseudomonas aeruginosa* were important bacteria leading to mortality in elderly SAE patients (Singer et al., 2016). The mechanism of sepsis caused by *Klebsiella pneumoniae* infection is complex, mainly through its virulence factors such as the capsule, which inhibits macrophage function, resulting in difficult infection control (Togawa et al., 2015). The rapid multiplication of bacteria releases toxins and inflammatory mediators, triggering a systemic inflammatory response that further leads to multi-organ dysfunction (Wei et al., 2014), especially, when the infection spreads to the brain, which can lead to SAE and significantly increase patient mortality, based on clinical data analysis, this study explores the effect of *Klebsiella pneumoniae* infection on mortality from SAE in the elderly patients. Studies had found that *Klebsiella pneumoniae* infection significantly increases the mortality rate of elderly SAE patients, and the mechanism may be related to the severe inflammatory response and BBB damage caused by the bacterium (Lippmann et al., 2014). Clinical attention should be paid to the management of *Klebsiella pneumoniae* infection to reduce the mortality rate of SAE. In addition, this study found that *Pseudomonas aeruginosa* infection significantly increased mortality in older sepsis patients and SAE. The bacterium is highly resistant to drugs due to the presence of 16S rRNA methylases of the armA gene family (Aghazadeh et al., 2013), and it is difficult to treat after infection, which can easily lead to deterioration of the disease and multi-organ failure, especially the damage to the nervous system. SAE is more common and more dangerous in sepsis patients with *Pseudomonas aeruginosa* infection, directly increasing the risk of death. Therefore, effective control of bacterial infections with *Pseudomonas aeruginosa* and *Klebsiella pneumoniae* requires a broader, more robust team that encompasses medicine, nursing, infection control, environmental health, and patient and family education. To effectively control the infection of these two bacteria through multidisciplinary collaboration, doctors conduct bacterial culture and antimicrobial susceptibility tests to inform the selection of appropriate antibiotics. The care team is responsible for the daily care of the patient, including monitoring vital signs, administering medications, turning over and patting the back, etc., to reduce the risk of infection. He is also responsible for supervising and enforcing the hospital’s infection control policies, such as hand hygiene, environmental disinfection, etc. In summary, through the collaboration of multidisciplinary teams and the implementation of integrated strategies, the bacterial infection of *Pseudomonas aeruginosa* and *Klebsiella pneumoniae* can be effectively controlled, the rate of nosocomial infection can be reduced, and the quality of life of patients can be improved.

This study deeply analyzed the prognostic factors of elderly sepsis patients with encephalopathy, and provided important enlightenment for clinical practice. Which is recommended that clinicians should strengthen the early and dynamic monitoring of SAPS III score, APTT and lactate level changes in elderly patients with sepsis, timely correction of coagulation function, maintenance of effective tissue perfusion, regular monitoring of etiological changes, especially *Klebsiella pneumoniae* and *Pseudomonas aeruginosa*, and sensitive antibiotic treatment regimens according to drug susceptibility tests. At the same time, attention should be paid to the protection of multi-organ function, especially the monitoring and support of brain function, to improve the prognosis. In addition, strengthening interdisciplinary cooperation, formulating individualized treatment plans, and improving the overall level of diagnosis and treatment are the critical to improving the survival rate and quality of life of elderly patients with SAE.

### Limitations

This study discusses the limitations and future prospects of the study. The study was limited by relatively small sample sizes, the small sample size is susceptible to random variation, which may leads to increased chance of research results and is difficult to reflect the overall real situation. Besides, the small sample size may not adequately represent the characteristics of the population, making it difficult to generalize the findings to other populations, the results of the study may be influenced by the characteristics of a particular sample, limiting their external validity. In this study, the bias in retrospective analysis was avoided as effectively as possible by clarifying the study design, strictly selecting the study subjects, ensuring data quality, controlling confounding factors, and carefully interpreting the study results, but it did not exclude the potential bias caused by retrospective analysis, which affected the results of this study. Future studies should expand the sample size, adopt a multi-center, prospective design, and include more biomarkers and other in-depth explorations, so as to more comprehensively reveal the prognostic factors of elderly sepsis patients with pathogenic microbial-positive and SAE, and provide more precise treatment strategies and interventions for clinical practice. We consider that the mechanism of encephalopathy caused by *Klebsiella pneumonia* and *Pseudomonas aeruginosa* may be related to leaky BBB, however, validation of S100B protein in patients with SAE was lacking in this study, further validation of the mechanism will be required in the future (Malik et al., 2023). Although the results of this study suggest that *Klebsiella pneumonia* and *Pseudomonas aeruginosa* were independent risk factors for the prognosis of elderly patients with SAE, the potential bias caused by the detection accuracy of the two pathogens in the database cannot be ruled out.

## Conclusion

This study conducted an in-depth analysis of the prognosis of elderly sepsis patients with pathogenic microorganism-positive and SAE. Primary findings include the high mortality rate of SAE in older patients. *Klebsiella pneumoniae* and *Pseudomonas aeruginosa* infection, the high level of APTT and lactate and SAPS III score were the main factors contributing to poor prognosis. These findings provide an important reference for the treatment and care of clinically elderly sepsis patients with SAE.

## Data Availability

Publicly available datasets were analyzed in this study. This data can be found here: the data source of this study is the publicly available MIMIC IV database, https://mimic.mit.edu.
