# Supplementary material for: Prognostic analysis of elderly patients with pathogenic microorganisms positive for sepsis-associated encephalopathy
Source: Front Microbiol. 2024 Dec 16;15:1509726. doi: 10.3389/fmicb.2024.1509726 (PMC11718444; doi:10.3389/fmicb.2024.1509726)
Supplement: Supplementary file 1 [file Data_Sheet_1.docx]

| **Supplementary Material 1 Baseline and outcome of young patients for sepsis-associated encephalopathy** | | | |
| --- | --- | --- | --- |
| **Characteristic** | **Survival group(n=541)** | **Non-Survival group(n=51)** | ***P*** |
| Age, years | 54.00 [47.00, 61.00] | 58.00 [49.00, 62.00] | 0.213 |
| Male sex, n (%) | 317 (58.6) | 29 ( 56.9) | 0.927 |
| **Co-morbid conditions, n (%)** | | | |
| Charlson | 3.00 [2.00, 5.00] | 5.00 [3.00, 7.00] | <0.001 |
| Hypertension | 232 (42.9) | 19 ( 37.3) | 0.529 |
| Diabetes | 145 (26.8) | 13 ( 25.5) | 0.971 |
| Chronic obstructive pulmonary disease | 101 (18.7) | 8 ( 15.7) | 0.737 |
| Chronic kidney diseae | 88 (16.3) | 12 ( 23.5) | 0.259 |
| **Site of infection, n (%)** | | | |
| Pulmonary infection | 46 ( 8.5) | 6 ( 11.8) | 0.598 |
| Abdominal infection | 29 ( 5.4) | 2 ( 3.9) | 0.911 |
| Urinary infection | 37 ( 6.8) | 0 ( 0.0) | 0.104 |
| Skin softtissue infection | 28 ( 5.2) | 2 ( 3.9) | 0.955 |
| Catheter infection | 23 ( 4.3) | 2 ( 3.9) | 1.000 |
| **Pathogenic microorganisms, n (%)** | | | |
| Acinetobacter baumannii | 12 ( 2.2) | 1 ( 2.0) | 1.000 |
| Klebsiellapneumoniae | 79 (14.6) | 4 ( 7.8) | 0.264 |
| Pseudomonas aeruginosa | 55 (10.2) | 2 ( 3.9) | 0.231 |
| Staphylococcus aureus | 41 ( 7.6) | 1 ( 2.0) | 0.227 |
| Escherichiacoli | 103 (19.0) | 2 ( 3.9) | 0.012 |
| **Physiology** | | | |
| Temperature,℃ | 37.39 [37.06, 37.83] | 37.22 [36.75, 37.67] | 0.113 |
| Heart rate,beats per minute | 94.00 [81.00, 106.00] | 88.00 [69.00, 109.50] | 0.203 |
| Systolicblood pressure, mmHg | 107.00 [94.00, 127.00] | 103.00 [88.00, 122.50] | 0.137 |
| Diastolicblood pressure,mmHg | 59.50 [50.00, 70.00] | 57.00 [42.50, 66.00] | 0.037 |
| Respiratory rate,beats per minute | 22.00 [17.00, 27.00] | 21.00 [17.00, 26.75] | 0.760 |
| **Laboratory tests** | | | |
| **Blood system** | | | |
| White blood cell×109 /L | 13.50 [9.25, 17.95] | 17.10 [11.20, 22.35] | 0.002 |
| Hemoglobin(g/dL) | 9.60 [8.10, 11.45] | 9.40 [8.00, 11.00] | 0.498 |
| Platelet (×10ˆ9 /L) | 168.00 [109.50, 243.00] | 139.00 [77.50, 223.50] | 0.082 |
| PT(sec) | 14.70 [12.80, 18.80] | 18.80 [14.70, 26.25] | <0.001 |
| APTT(sec) | 34.20 [28.80, 44.78] | 44.78 [31.45, 55.50] | 0.001 |
| INR | 1.30 [1.20, 1.70] | 1.72 [1.35, 2.55] | <0.001 |
| **Other organ functions** | | | |
| Creatinine(mg/dL) | 1.00 [0.70, 1.60] | 1.40 [1.00, 2.90] | <0.001 |
| Bun(mg/dL) | 19.00 [13.00, 30.00] | 32.00 [16.00, 51.50] | <0.001 |
| Glucose(mg/dL) | 160.00 [126.00, 202.00] | 157.00 [130.50, 202.00] | 0.972 |
| Lactate (mmol/L) | 2.00 [1.30, 2.50] | 2.50 [1.75, 4.00] | 0.003 |
| PaCO_2_,mmHg | 40.00 [35.00, 45.00] | 39.00 [34.00, 45.00] | 0.451 |
| SpO_2_,% | 93.00 [91.00, 95.00] | 93.00 [91.00, 94.00] | 0.178 |
| **Treatment strategies** | | | |
| Use of vasoactive drugs, n (%) | 189 (34.9) | 35 ( 68.6) | <0.001 |
| Renal replacement therapy, n (%) | 34 ( 6.3) | 10 ( 19.6) | 0.001 |
| **Outcome** | | | |
| SOFA | 3.00 [2.00, 5.00] | 5.00 [3.00, 8.00] | <0.001 |
| SAPS II | 33.00 [24.00, 43.00] | 45.00 [36.00, 51.50] | <0.001 |
| SAPS III | 45.00 [33.00, 60.00] | 65.00 [49.50, 75.50] | <0.001 |
| MELD | 11.00 [8.00, 21.00] | 23.08 [10.50, 30.56] | <0.001 |
| LODS | 5.00 [3.00, 7.00] | 7.00 [5.00, 8.00] | <0.001 |
| OASIS | 31.00 [25.00, 37.00] | 36.00 [31.00, 39.00] | 0.001 |
| Los_icu | 4.12 [2.04, 10.13] | 6.39 [3.62, 9.53] | 0.069 |
| Los_hospital | 15.52 [7.46, 27.99] | 12.25 [7.35, 18.14] | 0.015 |

APPT: Activated partial thrombin time; BUN: blood urea nitrogen; INR: International Normalized Ratio; PT: Prothrombin time; SAPS: simplified acute physiology score; SOFA: Sequential organ failure assessment; LODS:Logical evaluation system for organ dysfunction; OASIS: Oxford acute severity of illness score; *P* < 0.05, statistically significant.

| **Supplementary Material 2 Baseline and outcome of Pathogenic microorganisms for sepsis-associated encephalopathy** | | | |
| --- | --- | --- | --- |
| **Characteristic** | **Other pathogenic microorganisms group(n=541)** | **Pseudomonas aeruginosa and Klebsiella pneumoniae group(n=233)** | ***P*** |
| Age, years | 77.00 [71.00, 84.00] | 79.00 [72.00, 85.00] | 0.033 |
| Male sex, n (%) | 413 ( 53.3) | 117 ( 50.2) | 0.453 |
| **Co-morbid conditions, n (%)** | | | |
| Charlson | 6.00 [4.00, 8.00] | 7.00 [5.00, 8.00] | 0.009 |
| Hypertension | 378 ( 48.8) | 130 ( 55.8) | 0.071 |
| Diabetes | 273 ( 35.2) | 94 ( 40.3) | 0.178 |
| Chronic obstructive pulmonary disease | 225 ( 29.0) | 64 ( 27.5) | 0.704 |
| Chronic kidney diseae | 227 ( 29.3) | 89 ( 38.2) | 0.013 |
| **Site of infection, n (%)** | | | |
| Pulmonary infection | 57 ( 7.4) | 36 ( 15.5) | <0.001 |
| Abdominal infection | 33 ( 4.3) | 16 ( 6.9) | 0.147 |
| Urinary infection | 72 ( 9.3) | 40 ( 17.2) | 0.001 |
| Skin softtissue infection | 46 ( 5.9) | 21 ( 9.0) | 0.133 |
| Catheter infection | 19 ( 2.5) | 15 ( 6.4) | 0.006 |
| **Physiology** | | | |
| Temperature,℃ | 37.17 [36.89, 37.56] | 37.11 [36.89, 37.56] | 0.492 |
| Heart rate,beats per minute | 89.00 [77.00, 104.50] | 92.00 [80.00, 110.00] | 0.015 |
| Systolicblood pressure, mmHg | 107.00 [90.00, 124.00] | 101.00 [88.00, 116.00] | 0.004 |
| Diastolicblood pressure,mmHg | 52.00 [44.00, 62.00] | 49.00 [40.50, 60.00] | 0.033 |
| Respiratory rate,beats per minute | 22.50 [18.00, 27.00] | 23.00 [18.00, 27.00] | 0.960 |
| **Laboratory tests** | | | |
| **Blood system** | | | |
| White blood cell×109 /L | 13.15 [9.30, 17.20] | 14.40 [9.90, 18.00] | 0.086 |
| Hemoglobin(g/dL) | 9.40 [7.97, 11.00] | 8.90 [7.60, 10.50] | 0.013 |
| Platelet (×10ˆ9 /L) | 177.00 [117.00, 233.25] | 174.00 [128.00, 248.00] | 0.331 |
| PT(sec) | 15.20 [12.90, 18.80] | 15.90 [13.60, 19.20] | 0.023 |
| APTT(sec) | 33.90 [29.10, 44.78] | 37.00 [30.70, 45.20] | 0.015 |
| INR | 1.40 [1.20, 1.72] | 1.50 [1.20, 1.72] | 0.028 |
| **Other organ functions** | | | |
| Creatinine(mg/dL) | 1.10 [0.80, 1.80] | 1.30 [0.90, 2.20] | 0.008 |
| Bun(mg/dL) | 25.00 [18.00, 40.00] | 29.00 [19.00, 46.00] | 0.006 |
| Glucose(mg/dL) | 162.00 [131.00, 204.75] | 164.00 [127.50, 211.00] | 0.622 |
| Lactate (mmol/L) | 2.10 [1.40, 2.50] | 2.00 [1.30, 2.50] | 0.462 |
| PaCO_2_,mmHg | 41.00 [35.00, 45.00] | 40.00 [35.00, 46.00] | 0.959 |
| SpO_2_,% | 92.00 [90.00, 95.00] | 93.00 [90.00, 95.00] | 0.263 |
| **Treatment strategies** | | | |
| Use of vasoactive drugs, n (%) | 19 ( 2.5) | 15 ( 6.4) | 0.006 |
| Renal replacement therapy, n (%) | 38 ( 4.9) | 14 ( 6.0) | 0.617 |
| **Outcome** | | | |
| SOFA | 3.00 [2.00, 5.00] | 3.00 [2.00, 4.00] | 0.792 |
| SAPS II | 41.00 [34.00, 50.00] | 46.00 [37.00, 54.00] | <0.001 |
| SAPS III | 47.00 [37.00, 60.00] | 52.00 [39.00, 64.00] | 0.009 |
| MELD | 13.00 [9.00, 20.00] | 16.00 [10.00, 22.00] | 0.004 |
| LODS | 5.00 [3.00, 7.00] | 5.00 [4.00, 8.00] | 0.025 |
| OASIS | 34.00 [29.00, 39.00] | 34.00 [28.00, 40.00] | 0.282 |
| Los_icu | 3.59 [1.94, 7.18] | 3.67 [1.85, 6.95] | 0.688 |
| Los_hospital | 11.68 [6.92, 19.96] | 13.10 [7.28, 23.52] | 0.072 |

APPT: Activated partial thrombin time; BUN: blood urea nitrogen; INR: International Normalized Ratio; PT: Prothrombin time; SAPS: simplified acute physiology score; SOFA: Sequential organ failure assessment; LODS:Logical evaluation system for organ dysfunction; OASIS: Oxford acute severity of illness score; *P* < 0.05, statistically significant.
