# Supplementary material for: Prognostic analysis of elderly patients with pathogenic microorganisms positive for sepsis-associated encephalopathy
Source: Front Microbiol. 2024 Dec 16;15:1509726. doi: 10.3389/fmicb.2024.1509726 (PMC11718444; doi:10.3389/fmicb.2024.1509726)
Supplement: Supplementary file 3 [file Table_4.DOCX]

**Reviewer 2**

C1: I thank the authors for their work entitled “Prognostic analysis of elderly patients with pathogenic microorganisms positive of sepsis-associated encephalopathy”. Instead of “of” it should be “for sepsis-associated….” The study focuses on the identification of pathogenic microbes for sepsis-associated encephalopathy. For the betterment of the manuscript following are my concerns/suggestions-

***R1: Thank you very much for your valuable comments. We have carefully reviewed and revised our manuscript based on your suggestions. This process has been insightful and has significantly enhanced the quality of our paper. Your comments have provided valuable guidance for both this revision and our future research efforts. We hope that the changes we have made align with your expectations and meet your approval. In addition, we have changed the "of" to "for" in manuscript on Page 1***

C2: The rationale of the study is not convincing. When there are known inflammatory responses, then why do authors think that these responses do not represent the host reaction to pathogenic microbes?

***R1: Thank you very much for your valuable comments. First of all: the inflammatory response is not triggered solely by pathogenic microorganisms. Inflammation can be caused by non-infectious causes, such as physical damage (e.g., mechanical trauma, burns, frostbite, etc.), chemical irritants (e.g., strong acids, bases, drugs, etc.), autoimmune diseases (autoantibodies attacking one's own tissues). Secondly: from the point of view of the specificity of the host response, The host response to pathogenic microorganisms is usually specific. When the immune system recognizes pathogenic microorganisms, it recognizes pathogen-related molecular patterns (PAMPs) through pattern recognition receptors (PRRs), such as toll-like receptors (TLRs), which recognize lipopolysaccharides (LPS) of bacteria. This recognition mechanism is capable of eliciting immune responses against specific pathogens, including innate and adaptive immunity. Some inflammatory responses may lack this specific recognition process for microorganisms. For example, in allergic inflammation, it is due to the body's hypersensitivity reaction to allergens (e.g., pollen, food, etc.), which is fundamentally different from the immune defense response against pathogenic microorganisms. The inflammatory process is mainly caused by IgE-mediated degranulation of mast cells, the release of inflammatory mediators such as histamine, and the mechanism of the immune response against pathogenic microorganisms is different.***

***Most importantly, pathogenic microorganisms are not detectable in all patients with sepsis in the sepsis population, and the positive rate of pathogenic microorganisms in patients with sepsis varies depending on a number of factors, including the sensitivity and specificity of the assay, the type of specimen, the type of pathogen infected, the stage of the disease, and the use of antimicrobials in the early stage, and in general, the positive rate may be around 30% to 40% when using traditional blood culture methods. However, with the use of more advanced molecular testing techniques (e.g., polymerase chain reaction, PCR), the test positivity rate can be increased to 50% to 70%. Especially, many previous studies had found that patients with sepsis infected with pathogenic microorganisms such as Klebsiella pneumoniae and Acinetobacter baumannii had a poor prognosis. Therefore, this study only explored sepsis populations that are positive for pathogenic microorganisms. We had made changes in the context of the study, which are as follows in page 1 and page 3:***

***Objectives: Sepsis-associated encephalopathy (SAE) has a high incidence and mortality, especially for elderly patients and patients who are positive for pathogenic microbial infection, this study explored the prognostic factors influencing the prognosis of elderly patients with pathogenic microorganisms positive of sepsis-associated encephalopathy.***

***In recent years, the research on SAE in elderly sepsis patients with pathogenic microorganisms infection have been increasingly deepened at home and abroad[5]. Especially, many previous studies had found that patients with sepsis infected with pathogenic microorganisms such as Klebsiella pneumoniae and Acinetobacter baumannii had a poor prognosis[6, 7]***

***[6]. Hosoda T, Harada S, Okamoto K, Ishino S, Kaneko M, Suzuki M, Ito R, Mizoguchi M: COVID-19 and Fatal Sepsis Caused by Hypervirulent Klebsiella pneumoniae, Japan, 2020. Emerging infectious diseases 2021, 27(2):556-559.***

***[7]. Marya, D., Zilberberg, Brian, H., Nathanson, Kate, Sulham, Weihong, Fan: Multidrug resistance, inappropriate empiric therapy, and hospital mortality in Acinetobacter baumannii pneumonia and sepsis. Critical Care 2016.***

C3. Control young group is missing because older people tend to harbor more microbes of a diverse range than young ones.

***R3: Thank you very much for your valuable comments. We divided the young group into the younger group and the older group for a comparative analysis in Table 1. Based on your recommendations, sepsis-associated encephalopathy in the younger group was further analysed and survival and non-survival groups were compared for analysis in Supplementary Material 1, the results of the study found that the site of infection and the type of infection of common pathogenic microorganisms in the ICU were not associated with the 28-day mortality rate in the young patient group. As follows:***

| **Supplementary Material 1 Baseline and outcome of young patients for sepsis-associated encephalopathy** | | | |
| --- | --- | --- | --- |
| **Characteristic** | **Survival group(n=775)** | **Non-Survival group(n=51)** | ***P*** |
| Age, years | 54.00 [47.00, 61.00] | 58.00 [49.00, 62.00] | 0.213 |
| Male sex, n (%) | 317 (58.6) | 29 ( 56.9) | 0.927 |
| **Co-morbid conditions, n (%)** | | | |
| Charlson | 3.00 [2.00, 5.00] | 5.00 [3.00, 7.00] | <0.001 |
| Hypertension | 232 (42.9) | 19 ( 37.3) | 0.529 |
| Diabetes | 145 (26.8) | 13 ( 25.5) | 0.971 |
| Chronic obstructive pulmonary disease | 101 (18.7) | 8 ( 15.7) | 0.737 |
| Chronic kidney diseae | 88 (16.3) | 12 ( 23.5) | 0.259 |
| **Site of infection, n (%)** | | | |
| Pulmonary infection | 46 ( 8.5) | 6 ( 11.8) | 0.598 |
| Abdominal infection | 29 ( 5.4) | 2 ( 3.9) | 0.911 |
| Urinary infection | 37 ( 6.8) | 0 ( 0.0) | 0.104 |
| Skin softtissue infection | 28 ( 5.2) | 2 ( 3.9) | 0.955 |
| Catheter infection | 23 ( 4.3) | 2 ( 3.9) | 1 |
| **Pathogenic microorganisms, n (%)** | | | |
| Acinetobacter baumannii | 12 ( 2.2) | 1 ( 2.0) | 1 |
| Klebsiellapneumoniae | 79 (14.6) | 4 ( 7.8) | 0.264 |
| Pseudomonas aeruginosa | 55 (10.2) | 2 ( 3.9) | 0.231 |
| Staphylococcus aureus | 41 ( 7.6) | 1 ( 2.0) | 0.227 |
| Escherichiacoli | 103 (19.0) | 2 ( 3.9) | 0.012 |
| **Physiology** | | | |
| Temperature,℃ | 37.39 [37.06, 37.83] | 37.22 [36.75, 37.67] | 0.113 |
| Heart rate,beats per minute | 94.00 [81.00, 106.00] | 88.00 [69.00, 109.50] | 0.203 |
| Systolicblood pressure, mmHg | 107.00 [94.00, 127.00] | 103.00 [88.00, 122.50] | 0.137 |
| Diastolicblood pressure,mmHg | 59.50 [50.00, 70.00] | 57.00 [42.50, 66.00] | 0.037 |
| Respiratory rate,beats per minute | 22.00 [17.00, 27.00] | 21.00 [17.00, 26.75] | 0.76 |
| **Laboratory tests** | | | |
| **Blood system** | | | |
| White blood cell×109 /L | 13.50 [9.25, 17.95] | 17.10 [11.20, 22.35] | 0.002 |
| Hemoglobin(g/dL) | 9.60 [8.10, 11.45] | 9.40 [8.00, 11.00] | 0.498 |
| Platelet (×10ˆ9 /L) | 168.00 [109.50, 243.00] | 139.00 [77.50, 223.50] | 0.082 |
| PT(sec) | 14.70 [12.80, 18.80] | 18.80 [14.70, 26.25] | <0.001 |
| APTT(sec) | 34.20 [28.80, 44.78] | 44.78 [31.45, 55.50] | 0.001 |
| INR | 1.30 [1.20, 1.70] | 1.72 [1.35, 2.55] | <0.001 |
| **Other organ functions** | | | |
| Creatinine(mg/dL) | 1.00 [0.70, 1.60] | 1.40 [1.00, 2.90] | <0.001 |
| Bun(mg/dL) | 19.00 [13.00, 30.00] | 32.00 [16.00, 51.50] | <0.001 |
| Glucose(mg/dL) | 160.00 [126.00, 202.00] | 157.00 [130.50, 202.00] | 0.972 |
| Lactate (mmol/L) | 2.00 [1.30, 2.50] | 2.50 [1.75, 4.00] | 0.003 |
| PaCO_2_,mmHg | 40.00 [35.00, 45.00] | 39.00 [34.00, 45.00] | 0.451 |
| SpO_2_,% | 93.00 [91.00, 95.00] | 93.00 [91.00, 94.00] | 0.178 |
| **Treatment strategies** | | | |
| Use of vasoactive drugs, n (%) | 189 (34.9) | 35 ( 68.6) | <0.001 |
| Renal replacement therapy, n (%) | 34 ( 6.3) | 10 ( 19.6) | 0.001 |
| **Outcome** | | | |
| SOFA | 3.00 [2.00, 5.00] | 5.00 [3.00, 8.00] | <0.001 |
| SAPS II | 33.00 [24.00, 43.00] | 45.00 [36.00, 51.50] | <0.001 |
| SAPS III | 45.00 [33.00, 60.00] | 65.00 [49.50, 75.50] | <0.001 |
| MELD | 11.00 [8.00, 21.00] | 23.08 [10.50, 30.56] | <0.001 |
| LODS | 5.00 [3.00, 7.00] | 7.00 [5.00, 8.00] | <0.001 |
| OASIS | 31.00 [25.00, 37.00] | 36.00 [31.00, 39.00] | 0.001 |
| Los_icu | 4.12 [2.04, 10.13] | 6.39 [3.62, 9.53] | 0.069 |
| Los_hospital | 15.52 [7.46, 27.99] | 12.25 [7.35, 18.14] | 0.015 |

APPT: Activated partial thrombin time; BUN: blood urea nitrogen; INR: International Normalized Ratio; PT: Prothrombin time; SAPS: simplified acute physiology score; SOFA: Sequential organ failure assessment; LODS:Logical evaluation system for organ dysfunction; OASIS: Oxford acute severity of illness score; P < 0.05, statistically significant.

C4: Sepsis-causing bacteria must be discussed.

***R4: Thank you very much for your valuable comments. Based on your comments, we discussed the bacteria that cause sepsis in this study in Page 10-11, As follows:***

Common pathogens that cause sepsis in intensive care medicine include: *Escherichia coli*, *Klebsiella pneumoniae* and *Pseudomonas aeruginosa* in gram-negative bacteria; Gram-positive bacteria include *Staphylococcus aureus*, the release of cell wall components and exotoxins from these bacteria can cause a systemic inflammatory response syndrome, leading to organ dysfunction, and the mortality rate of sepsis patients increases significantly as the number of organs affected. In this study, it was found that *Klebsiella pneumoniae* and *Pseudomonas aeruginosa* were important bacteria leading to mortality in elderly SAE patients[14]. The mechanism of sepsis caused by *Klebsiella pneumoniae* infection is complex, mainly through its virulence factors such as the capsule, which inhibits macrophage function, resulting in difficult infection control[23]. The rapid multiplication of bacteria releases toxins and inflammatory mediators, triggering a systemic inflammatory response that further leads to multi-organ dysfunction[24], especially, when the infection spreads to the brain, which can lead to SAE and significantly increase patient mortality, based on clinical data analysis, this study explores the effect of *Klebsiella pneumoniae* infection on mortality from SAE in the elderly patients. Studies had found that *Klebsiella pneumoniae* infection significantly increases the mortality rate of elderly SAE patients, and the mechanism may be related to the severe inflammatory response and BBB damage caused by the bacterium[25]. Clinical attention should be paid to the management of *Klebsiella pneumoniae* infection to reduce the mortality rate of SAE. In addition, this study found that *Pseudomonas aeruginosa* infection significantly increased mortality in older sepsis patients and SAE. The bacterium is highly resistant to drugs, and it is difficult to treat after infection, which can easily lead to deterioration of the disease and multi-organ failure, especially the damage to the nervous system. SAE is more common and more dangerous in sepsis patients with *Pseudomonas aeruginosa* infection, directly increasing the risk of death. Therefore, early recognition and effective treatment of *Pseudomonas aeruginosa* infection is essential to reduce mortality from encephalopathy associated with elderly sepsis patients.

C5:Experiments should also be performed for known sepsis causative agents like “staph” and “streps” in comparison with “KP” and ‘AE” bacteria.

***R5: Thank you very much for your valuable comments, based on your suggestion, we put sepsis causative agents like "Acinetobacter baumannii",”Staphylococcus aureus” and "Escherichiacoli" and other pathogenic bacteria in comparison with "KP" and 'AE" bacteria in Supplementary Material 2, the study found that patients with Pseudomonas aeruginosa and Klebsiella pneumoniae group had more severe disease severity and a worse prognosis, compared with other microbial infections, as follows:***

| **Supplementary Material 2 Baseline and outcome of Pathogenic microorganisms for sepsis-associated encephalopathy** | | | |
| --- | --- | --- | --- |
| **Characteristic** | **Other pathogenic microorganisms group(n=541)** | **Pseudomonas aeruginosa and Klebsiella pneumoniae group(n=233)** | ***P*** |
| Age, years | 77.00 [71.00, 84.00] | 79.00 [72.00, 85.00] | 0.033 |
| Male sex, n (%) | 413 ( 53.3) | 117 ( 50.2) | 0.453 |
| **Co-morbid conditions, n (%)** | | | |
| Charlson | 6.00 [4.00, 8.00] | 7.00 [5.00, 8.00] | 0.009 |
| Hypertension | 378 ( 48.8) | 130 ( 55.8) | 0.071 |
| Diabetes | 273 ( 35.2) | 94 ( 40.3) | 0.178 |
| Chronic obstructive pulmonary disease | 225 ( 29.0) | 64 ( 27.5) | 0.704 |
| Chronic kidney diseae | 227 ( 29.3) | 89 ( 38.2) | 0.013 |
| **Site of infection, n (%)** | | | |
| Pulmonary infection | 57 ( 7.4) | 36 ( 15.5) | <0.001 |
| Abdominal infection | 33 ( 4.3) | 16 ( 6.9) | 0.147 |
| Urinary infection | 72 ( 9.3) | 40 ( 17.2) | 0.001 |
| Skin softtissue infection | 46 ( 5.9) | 21 ( 9.0) | 0.133 |
| Catheter infection | 19 ( 2.5) | 15 ( 6.4) | 0.006 |
| **Physiology** | | | |
| Temperature,℃ | 37.17 [36.89, 37.56] | 37.11 [36.89, 37.56] | 0.492 |
| Heart rate,beats per minute | 89.00 [77.00, 104.50] | 92.00 [80.00, 110.00] | 0.015 |
| Systolicblood pressure, mmHg | 107.00 [90.00, 124.00] | 101.00 [88.00, 116.00] | 0.004 |
| Diastolicblood pressure,mmHg | 52.00 [44.00, 62.00] | 49.00 [40.50, 60.00] | 0.033 |
| Respiratory rate,beats per minute | 22.50 [18.00, 27.00] | 23.00 [18.00, 27.00] | 0.96 |
| **Laboratory tests** | | | |
| **Blood system** | | | |
| White blood cell×109 /L | 13.15 [9.30, 17.20] | 14.40 [9.90, 18.00] | 0.086 |
| Hemoglobin(g/dL) | 9.40 [7.97, 11.00] | 8.90 [7.60, 10.50] | 0.013 |
| Platelet (×10ˆ9 /L) | 177.00 [117.00, 233.25] | 174.00 [128.00, 248.00] | 0.331 |
| PT(sec) | 15.20 [12.90, 18.80] | 15.90 [13.60, 19.20] | 0.023 |
| APTT(sec) | 33.90 [29.10, 44.78] | 37.00 [30.70, 45.20] | 0.015 |
| INR | 1.40 [1.20, 1.72] | 1.50 [1.20, 1.72] | 0.028 |
| **Other organ functions** | | | |
| Creatinine(mg/dL) | 1.10 [0.80, 1.80] | 1.30 [0.90, 2.20] | 0.008 |
| Bun(mg/dL) | 25.00 [18.00, 40.00] | 29.00 [19.00, 46.00] | 0.006 |
| Glucose(mg/dL) | 162.00 [131.00, 204.75] | 164.00 [127.50, 211.00] | 0.622 |
| Lactate (mmol/L) | 2.10 [1.40, 2.50] | 2.00 [1.30, 2.50] | 0.462 |
| PaCO_2_,mmHg | 41.00 [35.00, 45.00] | 40.00 [35.00, 46.00] | 0.959 |
| SpO_2_,% | 92.00 [90.00, 95.00] | 93.00 [90.00, 95.00] | 0.263 |
| **Treatment strategies** | | | |
| Use of vasoactive drugs, n (%) | 19 ( 2.5) | 15 ( 6.4) | 0.006 |
| Renal replacement therapy, n (%) | 38 ( 4.9) | 14 ( 6.0) | 0.617 |
| **Outcome** | | | |
| SOFA | 3.00 [2.00, 5.00] | 3.00 [2.00, 4.00] | 0.792 |
| SAPS II | 41.00 [34.00, 50.00] | 46.00 [37.00, 54.00] | <0.001 |
| SAPS III | 47.00 [37.00, 60.00] | 52.00 [39.00, 64.00] | 0.009 |
| MELD | 13.00 [9.00, 20.00] | 16.00 [10.00, 22.00] | 0.004 |
| LODS | 5.00 [3.00, 7.00] | 5.00 [4.00, 8.00] | 0.025 |
| OASIS | 34.00 [29.00, 39.00] | 34.00 [28.00, 40.00] | 0.282 |
| Los_icu | 3.59 [1.94, 7.18] | 3.67 [1.85, 6.95] | 0.688 |
| Los_hospital | 11.68 [6.92, 19.96] | 13.10 [7.28, 23.52] | 0.072 |

APPT: Activated partial thrombin time; BUN: blood urea nitrogen; INR: International Normalized Ratio; PT: Prothrombin time; SAPS: simplified acute physiology score; SOFA: Sequential organ failure assessment; LODS:Logical evaluation system for organ dysfunction; OASIS: Oxford acute severity of illness score; *P* < 0.05, statistically significant.

C6: Why The 16S rRNA methylase belonging to the armA gemne family should not be considered for KP.

***R6: Thank you very much for your valuable comments, based on your review comments, we added it to the discussion. As follows:***

***In addition, this study found that Pseudomonas aeruginosa infection significantly increased mortality in older sepsis patients and SAE. The bacterium is highly resistant to drugs due to the presence of 16S rRNA methylases of the armA gene family[26], and it is difficult to treat after infection, which can easily lead to deterioration of the disease and multi-organ failure, especially the damage to the nervous system.***

C7. Why authors are not proposing a broader and more vigorous regiment that can effectively control KP and AE bacteria, too?

***R7: Thank you very much for your valuable comments, based on your review comments, we added it to the discussion on Page 11. As follows: effective control of bacterial infections with Pseudomonas aeruginosa and Klebsiella pneumoniae requires a broader, more robust team that encompasses medicine, nursing, infection control, environmental health, and patient and family education. In order to effectively control the infection of these two bacteria through multidisciplinary collaboration, doctors conduct bacterial culture and antimicrobial susceptibility tests to inform the selection of appropriate antibiotics. The care team is responsible for the daily care of the patient, including monitoring vital signs, administering medications, turning over and patting the back, etc., to reduce the risk of infection. He is also responsible for supervising and enforcing the hospital's infection control policies, such as hand hygiene, environmental disinfection, etc. In summary, through the collaboration of multidisciplinary teams and the implementation of integrated strategies, the bacterial infection of Pseudomonas aeruginosa and Klebsiella pneumoniae can be effectively controlled, the rate of nosocomial infection can be reduced, and the quality of life of patients can be improved.***

C8. A leaky BBB (PMID: 37997517) must be experimentally validated by analyzing the S100B protein in the relevant patient.

***R8: Thank you very much for your valuable comments, validation could not be completed due to the lack of S100B protein in this study, and we clarified this shortcoming in the manuscript limitations in Page 12. As follows:***

***Limitations***

***This study discusses the limitations and future prospects of the study. The study was limited by relatively small sample sizes, which may affect the general applicability of the results. In addition, the absence of some clinical data and the inherent limitations of retrospective analysis may introduce a certain amount of bias. Future studies should expand the sample size, adopt a multi-center, prospective design, and include more biomarkers and other in-depth explorations, so as to more comprehensively reveal the prognostic factors of elderly sepsis patients with pathogenic microbial-positive and SAE, and provide more precise treatment strategies and interventions for clinical practice. In addition, we consider that the mechanism of encephalopathy caused by Klebsiella pneumonia and Pseudomonas aeruginosa may be related to leaky BBB, however, validation of S100B protein in patients with SAE was lacking in this study, further validation of the mechanism will be required in the future[27].***
