# Supplementary material for: Prognostic analysis of elderly patients with pathogenic microorganisms positive for sepsis-associated encephalopathy
Source: Front Microbiol. 2024 Dec 16;15:1509726. doi: 10.3389/fmicb.2024.1509726 (PMC11718444; doi:10.3389/fmicb.2024.1509726)
Supplement: Supplementary file 4 [file Table_5.DOCX]

**Reviewer 3**

Query 1: The introduction presents basic background information on SAE and the elderly, but it lacks depth in explaining why these factors are of particular concern. For example, the authors mention high mortality but provide no concrete statistics or data from prior studies to support this claim.

***R1: Thank you very much for your valuable comments. Based on your comments, we further delved into the background and added relevant arguments in Page 3. As follows:***

***In recent years, the research on SAE in elderly sepsis patients have been increasingly deepened at home and abroad[5]. Especially, many previous studies had found that patients with sepsis infected with pathogenic microorganisms such as Klebsiella pneumoniae and Acinetobacter baumannii had a poor prognosis[6, 7]. The studies have shown that SAE has a high incidence and poor prognosis in elderly patients with sepsis, which seriously affects the quality of life and survival of patients, the mortality rate in patients with sepsis encephalopathy is about 50-70%. Long-term follow-up showed that about 45% of patients with sepsis had cognitive dysfunction such as inattention and memory loss 1 year after discharge, and 10% of patients did not improve 3 years after discharge, or even remained after 6 years, which seriously affected the quality of life of patients [8-11]. The current research focuses on mechanisms such as inflammatory response, neurotransmitter dysregulation, and blood-brain barrier damage, as well as exploring novel biomarkers and therapeutics to improve the prognosis of patients with SAE[12, 13]. However, there is a lack of exploratory studies on prognostic risk factors in elderly patients with pathogenic microorganisms positive and SAE.***

Query 2: There is no sufficient critical appraisal of existing literature on the relationship between specific pathogens and SAE prognosis, which weakens the rationale for the study. Readers would benefit from a discussion of what’s missing in previous research, particularly on the distinct impact of Klebsiella pneumoniae and Pseudomonas aeruginosa.

***R2: Thank you very much for your valuable comments. Based on your comments, we further delved into the background and added relevant arguments in Page 3. As follows:***

***especially, many previous studies had found that patients with sepsis infected with pathogenic microorganisms such as Klebsiella pneumoniae and Acinetobacter baumannii increased multi-organ impairment and mortality in sepsis[6, 7]. The studies have shown that SAE has a high incidence and poor prognosis in elderly patients with sepsis, which seriously affects the quality of life and survival of patients, the mortality rate in patients with sepsis encephalopathy is about 50-70%. Long-term follow-up showed that about 45% of patients with sepsis had cognitive dysfunction such as inattention and memory loss 1 year after discharge, which seriously affected the quality of life of patients [8-11]. The current research focuses on mechanisms such as inflammatory response, neurotransmitter dysregulation, and blood-brain barrier damage, as well as exploring novel biomarkers and therapeutics to improve the prognosis of patients with SAE[12, 13]. The relationship between the type of pathogenic microorganisms infection, in particular, the common of Klebsiella pneumoniae, Acinetobacter baumannii, Pseudomonas aeruginosa, etc and the prognosis of patients with SAE remain unclear.***

Query 3: The hypothesis is overly simplistic and lacks specificity. It states that the prognosis is related to pathogen type and infection site without explaining how this study intends to address these aspects differently than previous research.

***R3: Thank you very much for your valuable comments. Based on your comments, we further delved into the hypothesis, previous studies included all patients with sepsis-associated encephalopathy, both those who were positive for the pathogenic microorganisms and those who were not found to be infected with the pathogenic microorganisms. However, this study is only for patients with SAE who are positive for pathogenic microorganisms. The changes are as follows in Page 4:***

***The hypothesis is that the prognosis of elderly patients with SAE is closely related to the type of pathogenic microorganisms, the site of infection by analysis of SAE patients who are positive for pathogenic microorganisms, and it is expected to improve the prognosis of such patients by optimizing the treatment regimen and strengthening the management of the underlying diseases.***

Query 4: The patient selection criteria are vaguely defined, particularly regarding SAE diagnosis. The authors define SAE broadly with the Glasgow Coma Scale (GCS) <15 but do not provide justification or precedent for this threshold. It raises concerns about whether patients were adequately screened for SAE versus other cognitive impairments.

***R4: Thank you very much for your valuable comments. Based on your comments, we chose a GCS score of 15 < based on the definition of sepsis-associated encephalopathy and reference to previous high-quality studies,we have clarified this on page 4 of the manuscript, and the changes are as follows:***

***SAE as defined and with reference to previous studies, in this study, we defined SAE in the study as sepsis with a Glasgow Coma Scale (GCS) < 15 during ICU hospitalization, or they were diagnosed as: delirium, cognitive impairment, altered mental status according to the ICD-9 code, or medicating with haloperidol[3, 16, 17].***

Query 5: Critical information on how variables were extracted from the MIMIC-IV database is missing. It is unclear if the authors accounted for data inconsistencies or ensured the accuracy of pathogen identification in patients’ records, which can be problematic in retrospective studies.

***R5: Thank you very much for your valuable comments. Based on your comments, we supplemented the information extracted from patient variables in Page 5-6, the changes are as follows:***

***Data Collection***

***In this study, the clinical data of sepsis patients with pathogenic microorganisms positive were collected from the MIMIC IV database, including the basic information of the patients(age, male), the detection results of pathogenic microorganisms(Acinetobacter baumannii, Klebsiella pneumoniae, Pseudomonas aeruginosa, Pseudomonas aeruginosa, Staphylococcus aureus, Escherichia coli), the site of infection, comorbidities(hypertension, diabetes, chronic obstructive pulmonary disease, Chronic kidney disease), the worst value of vital signs and laboratory tests within 24 hours of admission, the worst disease severity score (such asof Sequential Organ Failure Assessment score(SOFA); ), Simplified Acute Physiology Score II(SAPS II), SAPS III, model for end-stage liver disease(MELD), Logistic Organ Dysfunction System (LODS), Oxford acute severity of illness score(OASIS) were recorded during ICU hospitalization. Besides, we searched for vasoactive drugs and renal replacement therapy during hospital stays, the prognostic indicators of ICU admission, length of hospitalization, 28-day mortality rate and 90-day mortality rate were recorded in this study. In this study, the entire data retrieval and integration were carried out using SQL language and R language.***

Query 6: The study lacks justification for the choice of statistical tests (Wilcoxon, COX regression, ROC) and fails to address potential multiple comparison bias, risking inflated associations and non-replicable results.

***R6: Thank you very much for your valuable comments. Based on your comments, we fully explain the rationale for choosing this statistical method and and to clarify the approach to the treatment of potential bias for multiple comparisons in Page 6, the changes are as follows:***

***Statistics***

***The continuous variables in this study were all skewed, which were presented as the interquartile range (IQR). Since the study does not satisfy the normal distribution, Tthe Wilcoxon rank-sum and Fisher's exact tests were used for the comparison of elderly patients with SAE versus non-elderly patients with SAE, and survival group patients versus non-survival group patients. To analyze the relationship between covariates and 28-day mortality in elderly patients with SAE, univariate and multivariate COX regression analyses were selected. Univariate and multivariate COX regression analysis were used to explore the independent risk factors for 28-day mortality in elderly patients with pathogenic microorganism-positive and SAE. The covariates explored were used to assess the prognostic performance of older patients with SAE, using ROC curves. The section assesses the discrimination of indicators by plotting the receiver operating characteristic (ROC) curve and calculating the area under the curve (AUC) to determine the predictive accuracy of biomarkers for the prognosis of elderly patients with pathogenic microorganism-positive and SAE. In order to avoid bias bias due to multiplicity, we applied Bonferroni for correction, and the P-value given in this study is the corrected P-value. The Kaplan-Meier (KM) curves were used to analyze the prognosis of mortality at 28 days and 90 days in elderly and non-elderly patients.***

Results

Query 7: The study reports sensitivity and specificity for pathogen-related mortality without confidence intervals, undermining robustness. AUC values (e.g., 0.681 for SAPS III) indicate moderate predictive power, yet the authors overstate their impact, potentially misleading readers.

***R7: Thank you very much for your valuable comments. Based on your comments, we increased the confidence intervals in Figure 2. In addition, we have revised descriptions such as SAPS III in the manuscript of Page9. As follows:***


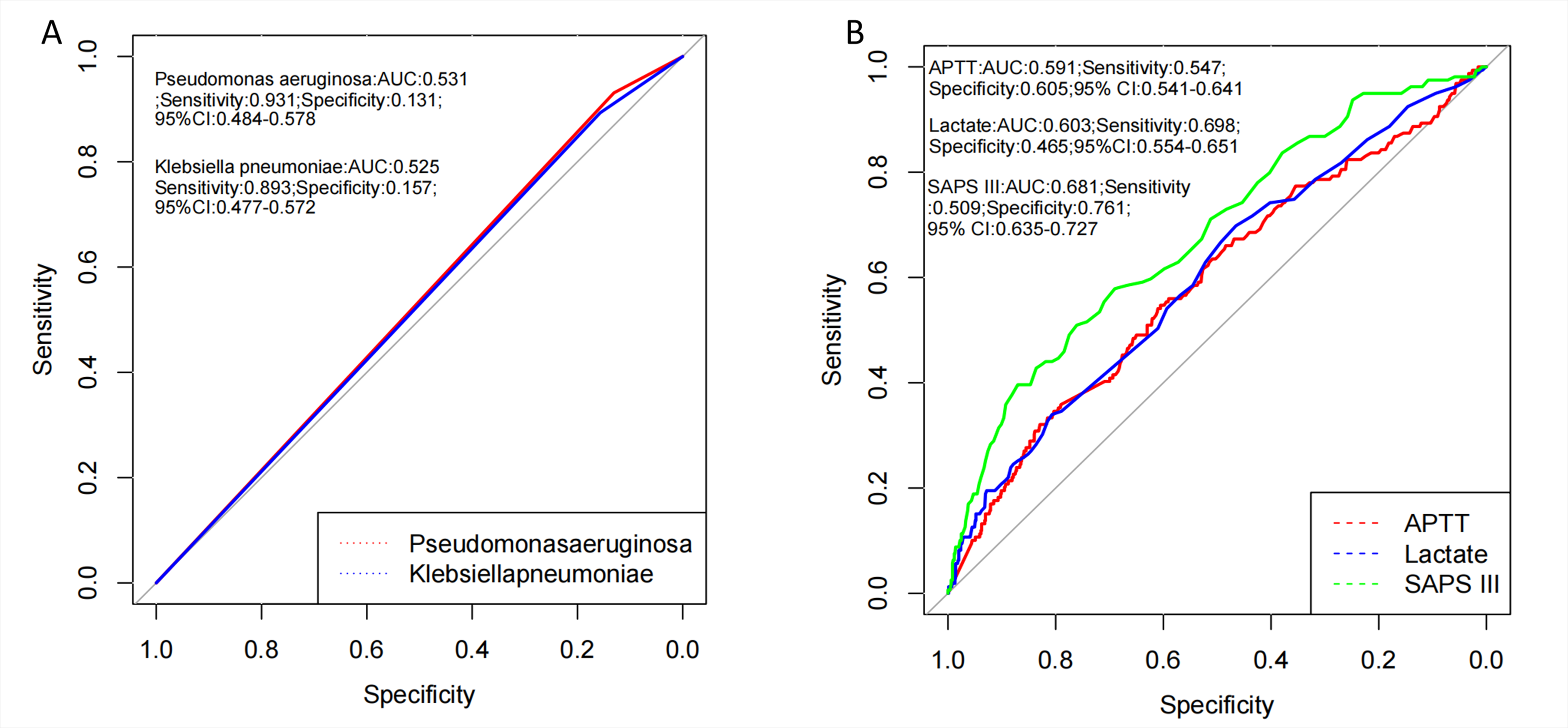


***Figure 2 shows the ROC curve, specificity, and sensitivity of Klebsiella pneumoniae and Pseudomonas aeruginosa infection, APTT and lactate, and SAPS III score for 28-day mortality in elderly SAE. The results of the study of Figure 2 shows that the AUC of Klebsiella pneumoniae, Pseudomonas aeruginosa, APTT and lactate, and SAPS III score respectively were 0.525, 0.531, 0.591, 0.603, 0.681; the sensitivity of Klebsiella pneumoniae, Pseudomonas aeruginosa, APTT and lactate, and SAPS III score respectively were 0.893, 0.931, 0.547, 0.698, 0.509; the specificity of Klebsiella pneumoniae, Pseudomonas aeruginosa, APTT and lactate, and SAPS III score respectively were 0.157, 0.131, 0.465, 0.605, 0.761; the AUC and specificity of SAPS III score was the better than other indicatorshighest AUC and specificity in the prognosis of elderly patients with pathogenic microorganisms positive and SAE, while the sensitive of Pseudomonas aeruginosa and Klebsiella pneumoniae infection were the better than other indicatorsmost sensitive.***

Query 8: Figures, particularly the Kaplan-Meier and ROC curves, are challenging to interpret without clearer legends. The authors should ensure all abbreviations and variable labels are fully explained. Some figures appear redundant, such as the comparison of APTT and lactate levels at different time points.

***R8: Thank you very much for your valuable comments. Based on your comments, we describe the legends in detail, so that the reader can interpret it. As follows:***

***Caption***

***Figure 1 Kaplan-Meier curves of 28-day and 90-day mortality in elderly and non-elderly sepsis-associated encephalopathy. Figure 1A: comparison of 28-day mortality in elderly versus non-elderly patients with SAE; Figure 1B: comparison of 90-day mortality in elderly versus non-elderly patients with SAE.***

***Figure 2 Analysis of the AUC values, specificity and sensitivity of Pseudomonas aeruginosa, Klebsiella pneumoniae, the levels of lactate, APTT, SAPS III scores in the area under the ROC curve of 28-day mortality in elderly sepsis patients with pathogenic microorganisms positive and SAE. Figure 2 shows that the AUC and specificity of SAPS III score was the better than other indicators in the prognosis of SAE, the sensitive of Pseudomonas aeruginosa and Klebsiella pneumoniae infection were the better than other indicators in the prognosis of SAE. SAPS III: Simplified Acute Physiology Score, APPT: Activated partial thrombin time.***

***Figure 3 The levels of lactate , APTT, and SAPS III scores were compared in 28-day versus 90-day mortality in elderly sepsis patients with pathogenic microorganisms positive and sepsis-associated encephalopathy. SAPS III: Simplified Acute Physiology Score, APPT: Activated partial thrombin time.***

Query 9: The discussion lacks a balanced interpretation. The authors highlight the prognostic value of Klebsiella pneumoniae and Pseudomonas aeruginosa without acknowledging the potential variability in pathogen detection accuracy in the MIMIC-IV database. This oversight raises questions about how generalizable their conclusions are to other cohorts.

***R9: Thank you very much for your valuable comments. Based on your comments, we illustrate the potential variability in pathogen detection accuracy in the MIMIC-IV database in the limitations in Page 14. As follows:***

***Limitations***

***This study discusses the limitations and future prospects of the study. The study was limited by relatively small sample sizes, which may affect the general applicability of the results. In addition, the absence of some clinical data and the inherent limitations of retrospective analysis may introduce a certain amount of bias. Future studies should expand the sample size, adopt a multi-center, prospective design, and include more biomarkers and other in-depth explorations, so as to more comprehensively reveal the prognostic factors of elderly sepsis patients with pathogenic microbial-positive and SAE, and provide more precise treatment strategies and interventions for clinical practice. We consider that the mechanism of encephalopathy caused by Klebsiella pneumonia and Pseudomonas aeruginosa may be related to leaky BBB, however, validation of S100B protein in patients with SAE was lacking in this study, further validation of the mechanism will be required in the future[29]. Although the results of this study suggest that Klebsiella pneumonia and Pseudomonas aeruginosa were independent risk factors for the prognosis of elderly patients with SAE, the potential bias caused by the detection accuracy of the two pathogens in the database cannot be ruled out.***

Query 10: The authors fail to position their results within the broader context of existing literature. Although they mention some studies, the comparison is superficial. For instance, they should compare mortality rates and risk factors identified in their study with those from other large-scale analyses of elderly sepsis patients with SAE.

***R10: Thank you very much for your valuable comments. Based on your and comments and Reviewer 2, we have revised the discussion section in page 10-14. As follows:***

***Discussion***

***This study conducted an in-depth analysis of the prognosis of elderly sepsis patients with pathogenic microorganism-positive and SAE. The results showed that the prognosis of elderly sepsis patients with SAE was generally poor, and their high mortality rate was closely related to the severity of SAE. Further analysis found that Klebsiella pneumoniae and Pseudomonas aeruginosa infection, the high level of APTT and lactate and SAPS III score were the main factors contributing to poor prognosis. The results of this study suggest that early identification and intervention of SAE and optimization of ICU management strategies were great significance for improving the prognosis of sepsis elderly patients.***

***Previous studies had found that sepsis-associated encephalopathy is as high as approximately 50-70%, the mortality rate of patients with sepsis who progress to SAE is about 10-50% [21, 24, 25].. The results of this study suggest that among the patients with sepsis-associated encephalopathy, 63% of the elderly patients with sepsis-associated encephalopathy, the mortality rate of elderly patients with SAE was significantly higher than that of non-elderly patients, this study found that the mortality rate of patients with SAE was about 20%, which is consistent with previous studies. Although specific morbidity and mortality vary depending on diagnostic criteria, underlying patient status, and treatment, the general consensus is that SAE significantly increases the risk of mortality and long-term cognitive impairment in older patients with sepsis[26, 27]. This study further emphasizes the importance of early screening, aggressive control of primary infection, and optimal management of sepsis in order to improve prognosis in sepsis older patients.***

***The prognosis of elderly patients with pathogenic microorganism-positive and SAE is multifactorial. Major independent risk factors include Klebsiella pneumoniae and Pseudomonas aeruginosa infection, the high level of APTT and lactate and SAPS III score, Klebsiella pneumoniae and Pseudomonas aeruginosa infection, the high level of APTT and lactate, and SAPS III score significantly increased the risk of mortality. Identification of these risk factors is helpful for early clinical intervention and optimization of treatment strategies, thereby improving the prognosis of elderly patients with SAE.***

***As a tool to predict the mortality rate of ICU patients, the SAPS III score also has important application value in patients with sepsis[28, 29]. The scoring system can accurately predict the mortality risk of patients with sepsis by comprehensively evaluating the physiological indicators, age and underlying diseases of patients, and provide a basis for clinical decision-making and medical resource allocation. Its simplicity and speed make the SAPS III score widely used in the initial evaluation and monitoring of patients with sepsis. In this section, a retrospective study found a significant correlation between SAPS III score and mortality in older patients with SAE. The SAPS III score can effectively predict the prognosis of elderly patients with pathogenic microorganisms positive and SAE, and the higher the score, the 28 days mortality rate with sepsis patients is significantly increased. These results suggest that the SAPS III score can be used as an important tool to clinically assess the severity and predict mortality of sepsis elderly patients with pathogenic microbial-positive and SAE.***

***Common pathogens that cause sepsis in intensive care medicine include: Escherichia coli, Klebsiella pneumoniae and Pseudomonas aeruginosa in gram-negative bacteria; Gram-positive bacteria include Staphylococcus aureus, the release of cell wall components and exotoxins from these bacteria can cause a systemic inflammatory response syndrome, leading to organ dysfunction, and the mortality rate of sepsis patients increases significantly as the number of organs affected. In this study, it was found that Klebsiella pneumoniae and Pseudomonas aeruginosa were important bacteria leading to mortality in elderly SAE patients[20]. The mechanism of sepsis caused by Klebsiella pneumoniae infection is complex, mainly through its virulence factors such as the capsule, which inhibits macrophage function, resulting in difficult infection control[30]. The rapid multiplication of bacteria releases toxins and inflammatory mediators, triggering a systemic inflammatory response that further leads to multi-organ dysfunction[31], especially, when the infection spreads to the brain, which can lead to SAE and significantly increase patient mortality, based on clinical data analysis, this study explores the effect of Klebsiella pneumoniae infection on mortality from SAE in the elderly patients. Studies had found that Klebsiella pneumoniae infection significantly increases the mortality rate of elderly SAE patients, and the mechanism may be related to the severe inflammatory response and BBB damage caused by the bacterium[32]. Clinical attention should be paid to the management of Klebsiella pneumoniae infection to reduce the mortality rate of SAE. In addition, this study found that Pseudomonas aeruginosa infection significantly increased mortality in older sepsis patients and SAE. The bacterium is highly resistant to drugs due to the presence of 16S rRNA methylases of the armA gene family[33], and it is difficult to treat after infection, which can easily lead to deterioration of the disease and multi-organ failure, especially the damage to the nervous system. SAE is more common and more dangerous in sepsis patients with Pseudomonas aeruginosa infection, directly increasing the risk of death. Therefore, effective control of bacterial infections with Pseudomonas aeruginosa and Klebsiella pneumoniae requires a broader, more robust team that encompasses medicine, nursing, infection control, environmental health, and patient and family education. To effectively control the infection of these two bacteria through multidisciplinary collaboration, doctors conduct bacterial culture and antimicrobial susceptibility tests to inform the selection of appropriate antibiotics. The care team is responsible for the daily care of the patient, including monitoring vital signs, administering medications, turning over and patting the back, etc., to reduce the risk of infection. He is also responsible for supervising and enforcing the hospital's infection control policies, such as hand hygiene, environmental disinfection, etc. In summary, through the collaboration of multidisciplinary teams and the implementation of integrated strategies, the bacterial infection of Pseudomonas aeruginosa and Klebsiella pneumoniae can be effectively controlled, the rate of nosocomial infection can be reduced, and the quality of life of patients can be improved.***

***.***

***This study deeply analyzed the prognostic factors of elderly sepsis patients with encephalopathy, and provided important enlightenment for clinical practice. Which is recommended that clinicians should strengthen the early and dynamic monitoring of SAPS III score, APTT and lactate level changes in elderly patients with sepsis, timely correction of coagulation function, maintenance of effective tissue perfusion, regular monitoring of etiological changes, especially Klebsiella pneumoniae and Pseudomonas aeruginosa, and sensitive antibiotic treatment regimens according to drug susceptibility tests.. At the same time, attention should be paid to the protection of multi-organ function, especially the monitoring and support of brain function, to improve the prognosis. In addition, strengthening interdisciplinary cooperation, formulating individualized treatment plans, and improving the overall level of diagnosis and treatment are the critical to improving the survival rate and quality of life of elderly patients with SAE.***

***Limitations***

***This study discusses the limitations and future prospects of the study. The study was limited by relatively small sample sizes, the small sample size is susceptible to random variation, which may leads to increased chance of research results and is difficult to reflect the overall real situation. Besides, the small sample size may not adequately represent the characteristics of the population, making it difficult to generalize the findings to other populations, the results of the study may be influenced by the characteristics of a particular sample, limiting their external validity. In this study, the bias in retrospective analysis was avoided as effectively as possible by clarifying the study design, strictly selecting the study subjects, ensuring data quality, controlling confounding factors, and carefully interpreting the study results, but it did not exclude the potential bias caused by retrospective analysis, which affected the results of this study. Future studies should expand the sample size, adopt a multi-center, prospective design, and include more biomarkers and other in-depth explorations, so as to more comprehensively reveal the prognostic factors of elderly sepsis patients with pathogenic microbial-positive and SAE, and provide more precise treatment strategies and interventions for clinical practice. We consider that the mechanism of encephalopathy caused by Klebsiella pneumonia and Pseudomonas aeruginosa may be related to leaky BBB, however, validation of S100B protein in patients with SAE was lacking in this study, further validation of the mechanism will be required in the future[34]. Although the results of this study suggest that Klebsiella pneumonia and Pseudomonas aeruginosa were independent risk factors for the prognosis of elderly patients with SAE, the potential bias caused by the detection accuracy of the two pathogens in the database cannot be ruled out.***

***Conclusion***

***This study conducted an in-depth analysis of the prognosis of elderly sepsis patients with pathogenic microorganism-positive and SAE. Primary findings include the high mortality rate of SAE in older patients. Klebsiella pneumoniae and Pseudomonas aeruginosa infection, the high level of APTT and lactate and SAPS III score were the main factors contributing to poor prognosis. These findings provide an important reference for the treatment and care of clinically elderly sepsis patients with SAE.***

Query 11: While the authors suggest the importance of early monitoring and intervention, they do not offer concrete guidelines or propose a clear management approach based on their findings. Readers would benefit from a more directive recommendation, such as the frequency of monitoring for specific risk factors or which biomarkers might warrant immediate intervention.

***R11: Thank you very much for your valuable comments. Based on your and comments, we made the following changes to the discussion as early as page 14.***

***This study deeply analyzed the prognostic factors of elderly sepsis patients with encephalopathy, and provided important enlightenment for clinical practice. Which is recommended that clinicians should strengthen the early and dynamic monitoring of SAPS III score, APTT and lactate level changes in elderly patients with sepsis, timely correction of coagulation function, maintenance of effective tissue perfusion, regular monitoring of etiological changes, especially Klebsiella pneumoniae and Pseudomonas aeruginosa, and sensitive antibiotic treatment regimens according to drug susceptibility tests.. At the same time, attention should be paid to the protection of multi-organ function, especially the monitoring and support of brain function, to improve the prognosis. In addition, strengthening interdisciplinary cooperation, formulating individualized treatment plans, and improving the overall level of diagnosis and treatment are the critical to improving the survival rate and quality of life of elderly patients with SAE.***

Query 12: The authors acknowledge a small sample size as a limitation but do not quantify its impact on statistical power or generalizability. Additionally, the lack of prospective data collection is briefly mentioned, but its implications for data accuracy and temporal bias are not fully explored.

***R12: Thank you very much for your valuable comments. Based on your and comments,we make changes to the limitations in Page 14. As follows:***

***Limitations***

***This study discusses the limitations and future prospects of the study. The study was limited by relatively small sample sizes, the small sample size is susceptible to random variation, which may leads to increased chance of research results and is difficult to reflect the overall real situation. Besides, the small sample size may not adequately represent the characteristics of the population, making it difficult to generalize the findings to other populations, the results of the study may be influenced by the characteristics of a particular sample, limiting their external validity.***

Query 13: The retrospective nature of the study introduces inherent biases that could be better addressed. The authors could have implemented strategies, such as sensitivity analyses, to mitigate some of these biases.

***R13: Thank you very much for your valuable comments. In this study, the bias in retrospective analysis was avoided as effectively as possible by clarifying the study design, strictly selecting the study subjects, ensuring data quality, controlling confounding factors, and carefully interpreting the study results, but it did not exclude the potential bias caused by retrospective analysis, which affected the results of this study. This issue was clarified in limitations of manuscript.***

Query 14: The references are relevant but lack recent studies from 2023 and 2024, which would strengthen the manuscript’s foundation. Ensuring that highly relevant, recent studies on SAE prognostic factors are included is essential.

***R14: Thank you very much for your valuable comments. We updated the reference lists for 2023 and 2024.***

Recommendation

The manuscript addresses an important topic, but it suffers from several limitations in methodology, statistical rigor, and depth of interpretation. While the study offers potentially useful data on SAE prognosis in elderly patients, its contribution to clinical knowledge is undermined by insufficient attention to biases, a lack of comparison with existing literature, and an overly simplistic discussion. To improve, the authors should refine their methodology, conduct more robust statistical analyses, and provide clearer guidance on clinical implications. Therefore, this manuscript needs major revision.

***R15: Thank you very much for your valuable comments. We have carefully reviewed and revised our manuscript based on your suggestions. This process has been insightful and has significantly enhanced the quality of our paper. Your comments have provided valuable guidance for both this revision and our future research efforts. We hope that the changes we have made align with your expectations and meet your approval.***
